# Bibliometric study on the utilization of sorafenib in hepatocellular carcinoma

**DOI:** 10.3389/fonc.2024.1507608

**Published:** 2024-12-20

**Authors:** Wenjun Meng, Yihang Luo, Lu Zhao, Yaoyu Zhang, Jiyan Liu, Shadan Li, Yang Du, Hongshuai Li

**Affiliations:** ^1^ Department of Biotherapy, Cancer Center, West China Hospital, Sichuan University, Chengdu, China; ^2^ Department of Urology, The General Hospital of Western Theater Command, Chengdu, China

**Keywords:** sorafenib, hepatocellular carcinoma, liver cancer, apoptosis, bibliometric analysis

## Abstract

**Background:**

Although the number of studies on sorafenib for hepatocellular carcinoma (HCC) is increasing during the past two decades, no detailed scientometric examination of its knowledge framework has been undertaken. Therefore, we performed a bibliometric analysis on this topic.

**Methods:**

VOSviewer and CiteSpace were utilized to analyze the articles regarding sorafenib for HCC from 2005 to 2024, which were retrieved from the Web of Science Core Collection (WoSCC) database.

**Results:**

There were 7,667 articles related to sorafenib in HCC were retrieved from the WoSCC database, and they covered 99 countries/regions, 5,640 institutions, and 30,450 authors. The most published literature of countries and institutions were China and Sun Yat-sen University, respectively. Cancers is the journal with the most papers published in this field, and the journal with the most co-citations is N Engl J Med. Among authors, Masatoshi Kudo has published the most research papers, and the most co-citations go to JM Llovet. The keywords “survival”, “apoptosis”, “efficacy”, “transarterial chemoembolization”, “lenvatinib”, etc. represent the current hotspots in this field.

**Conclusions:**

We identified current hotspots and trends by bibliometric analysis in sorafenib-HCC field, which might provide valuable guidance for future researches. Further explorations are supposed to conduct the continued study of HCC apoptosis, large-scaled clinical trials with international cooperations, and comprehensive treatments including multiple systemic or locoregional approaches in patients with HCC.

## 1 Introduction

Among all malignant tumors, the global morbidity and mortality rates of primary liver cancer rank sixth and third, respectively (1). The highest incidence of primary liver cancer in the world go to Asia and Africa, while the number in China accounts for about half of the global patients (2). Nearly 90% of patients with primary liver cancer are the type of hepatocellular carcinoma (HCC) (3). Although the incidence of HCC has a decreasing tendency in most countries due to the successful prevention of Hepatitis virus and aflatoxin, its poor prognosis reveals the slow process in the HCC treatments (2).

Molecular targeted drugs, including sorafenib, lenvatinib, regorafenib, etc., can intervene in key targets of the pathophysiological occurrence and development of HCC (3). In 2007, the tyrosine kinase inhibitor (TKI), sorafenib was authorized for the first systemic therapeutic drug in the first-line advanced HCC patients with Child-Pugh A, which was verified by multiple international trials (4). As the oral TKI drug, sorafenib can induce HCC cell apoptosis by promoting autophagy, reduce its proliferation, growth, and angiogenesis (5, 6). Due to the limited efficacy of the single drug, and the developing method of comprehensive treatment in cancer therapy, the combination of two or more therapeutic approaches prolongs patients’ survival (7). For example, while only about 30% advanced HCC are sensitive to sorafenib, more excellent results were observed when sorafenib combined immunotherapy, transarterial chemotherapy, or radiofrequency ablation (8, 9). Therefore, the use of sorafenib has also gradually converted during this growing trend.

Although the number of studies on sorafenib for HCC is increasing during the past two decades, no detailed scientometric examination of its knowledge framework has been undertaken. Given the special and important status of sorafenib in HCC, we performed this bibliometric study. Bibliometric studies are renowned for their ability to provide comprehensive insights into research trends and productivity through structured analyses of citation patterns, publication outputs, and collaboration networks. These analyses enable the identification of emerging research areas and critical gaps, fostering informed decision-making in academic and policy contexts (10). The robustness of bibliometric methodologies, often enhanced by advanced statistical and computational tools, ensures reliable assessments of research impact and visibility. Furthermore, the systematic evaluation of publication and citation data provides a dynamic framework for assessing institutional and individual research contributions, underscoring the strength of bibliometric approaches in understanding the evolution of scientific knowledge. Such methodological rigor not only quantifies academic productivity but also aids in recognizing significant contributors and their influence within various research domains (11).

In this study, a scientometric evaluation was firstly performed regarding the literature on sorafenib for HCC, utilizing bibliometric indicators to appraise the scholarly contributions, influence, and partnerships, pinpoint burgeoning areas of interest, and explore prospective directions for this domain.

## 2 Materials and methods

### 2.1 Data sources and searching strategies

The accuracy of document type annotation in the Web of Science Core Collection (WoSCC) database is better than any other database and is considered the best choice for literature analysis, so we chose to search in this database. We searched for all articles related to sorafenib in hepatocellular carcinoma research in WoSCC on September 1, 2024, using the following search formula: (((((((TS=(Sorafenib)) OR TS=("BAY 43-9006")) OR TS=("BAY 439006")) OR TS=("BAY 43 9006")) OR TS=("Sorafenib Tosylate")) OR TS=(Nexavar)) OR TS=("BAY 5459085")) OR TS=("BAY-673472") AND ((((TS=("Carcinomas, Hepatocellular")) OR TS=("Hepatocellular Carcinomas")) OR TS=("Hepatocellular Carcinoma")) OR TS=("Cell Carcinoma, Liver")) OR TS=("Liver Cell Carcinomas").

The inclusion criteria for literature screening were as follows: (1) full-text publications related to sorafenib in HCC; and (2) written in English. The exclusion criteria were as follows: (1) the topic was not related to sorafenib and HCC; (2) the paper type was review, meeting abstract, case report, letter, etc. Then the plain text version of the literature was exported. Because sorafenib has a long history of utilization and has been studied extensively, we excluded non-article type of literature to make the included literature more instructive.

### 2.2 Data extraction and analysis

Graphpad prism 8.0.2 was used to analyze and plot the annual papers and national paper publishing trends and proportions. CtieSpace 6.2.4R and VOSviewer 1.6.18 were used to analyze these data and visualize the scientific knowledge map.

**Figure 1:**
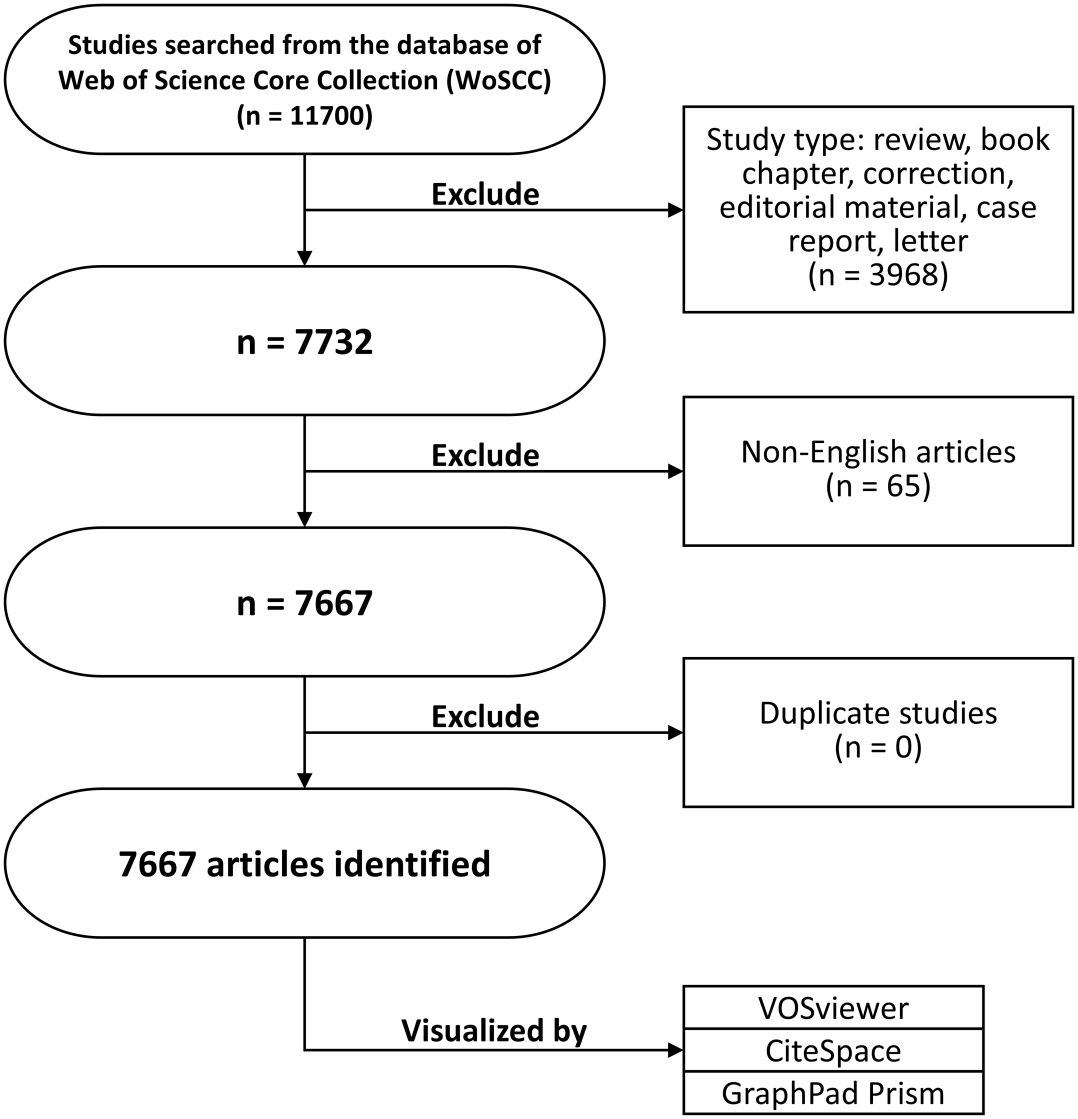
The flowchart of this study.

VOSviewer 1.6.18 was created by Waltman et al. in 2009. It is a JAVA-based free software for analyzing large amounts of literature data and displaying them in a map format. To visualize the research results in a certain field by drawing a co-citation network diagram of the literature, Professor Chaomei Chen created the CiteSpace software, which envisions using an experimental framework to study new concepts and evaluate existing technologies. This enables users to better understand knowledge areas, research frontiers and trends, and predict their future research progress. The flowchart of literature search is shown in **Figure 1**.

## 3 Results

In the WoSCC database, there were 7,667 articles related to sorafenib in HCC retrieved. They covered 99 countries or regions, 5,640 institutions, and 30,450 authors.

**Figure 2:**
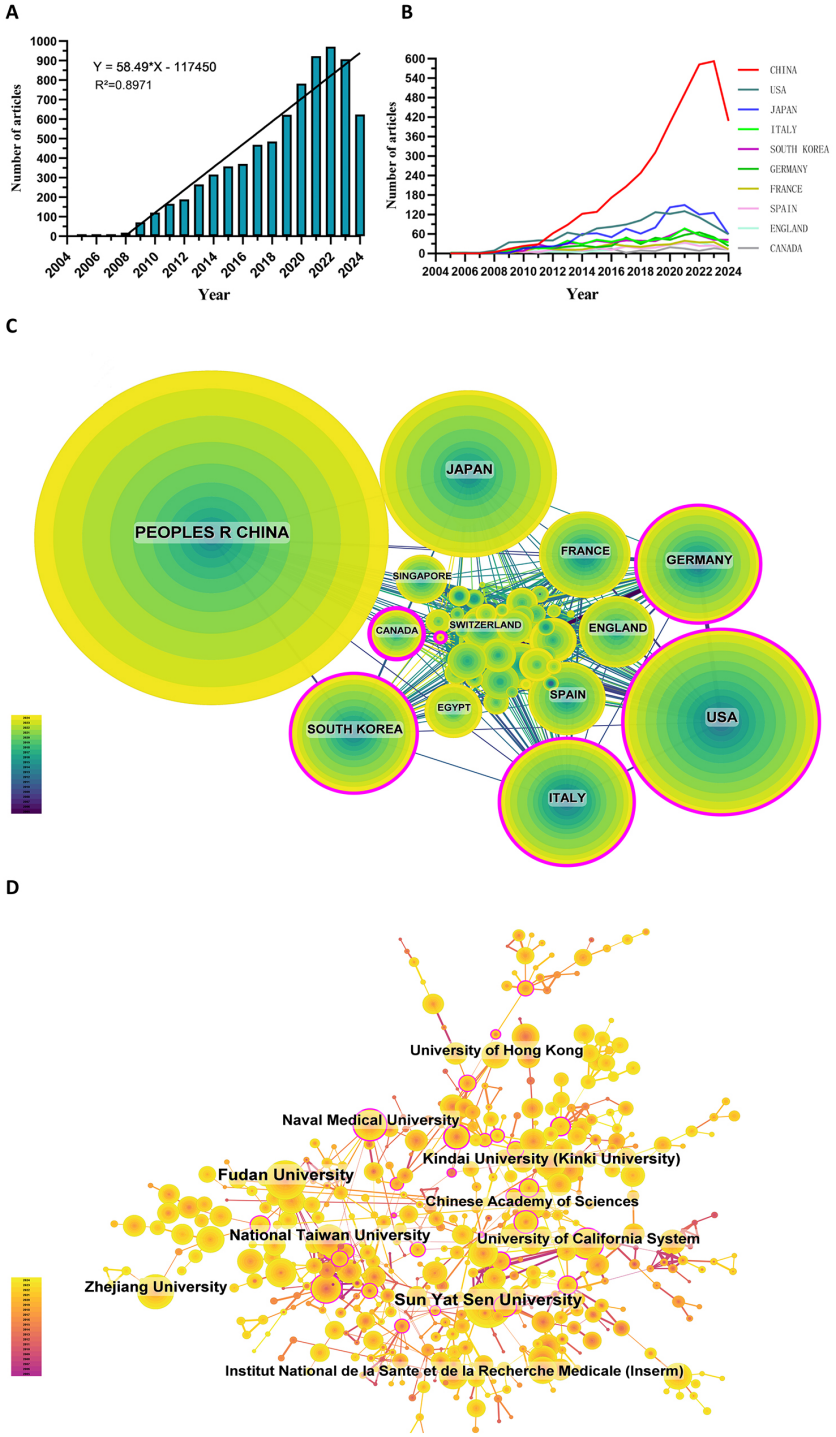
**(A)** Annual volume of publications. **(B)** Line graph of national publications. **(C)** Networks of country cooperation. **(D)** Networks of institutional co-operation.

Since 2005, the number of related publications has gradually increased. We divided it into three stages: (1) from 2005 to 2008, the growth was slow, and the annual number of publications was less than 20, indicating that the field developed slowly; (2) from 2009 to 2019, the publication volume increased rapidly; and (3) after 2020, the growth rate of the number increased further and reached the highest in 2022 (**Figure 2A**).

### 3.1 Countries/regions and institutions

To date, 99 countries/regions have conducted researches on sorafenib in HCC. **Figures 2B** show the annual publication volume of the top 10 countries in the past decades. The top 5 countries in this field are China, the United States, Japan, Italy and South Korea. China's paper number accounts for 50.66% of the total volume, far exceeding other countries.

Among the top ten countries/regions regarding paper publications, China's papers were cited 127,077 times, far exceeding all other countries/regions (**Table 1**). However, China’s paper citation/number ratio (32.72) ranks 10th among all countries, indicating that the published papers’ quality may be generally low. The United States ranks second in terms of the number of papers published (1,268), the number of citations (106,937 times) ranks second, and its citation/publication ratio (84.34) ranks high.

**Table 1:** Top ten countries’ published literature.

Rank	Country/region	Article counts (n=7,677, %)	Centrality	Citation	Citation per publication
1	China	3,884 (50.66)	0.04	127,077	32.72
2	USA	1,268 (16.54)	0.16	106,937	84.34
3	Japan	1,074 (14.01)	0.01	56,671	52.77
4	Italy	595 (7.76)	0.1	45,329	76.18
5	South Korea	582 (7.59)	0.13	45,998	79.03
6	Germany	518 (6.76)	0.12	44,503	85.91
7	France	332 (4.33)	0.07	43,735	131.73
8	Spain	250 (3.26)	0.05	41,249	165.00
9	England	248 (3.23)	0.09	26,834	108.20
10	Canada	145 (1.89)	0.29	19,333	133.33

The cooperation network is shown in **Figure 2C**. The United States has close cooperation with Germany, Italy, the United Kingdom and Spain, while China is closely cooperated with Japan, South Korea, Canada and Singapore. China has a large number of papers published and a high frequency of citations, indicating the leading country in this field.

There are 5,640 institutions systematically having published articles related to sorafenib in HCC. Among the top 10 institutions regarding paper publications, seven are from China, one from the United States, one from France, and one from Japan (**Table 2**, **Figure 2D**). Sun Yat-sen University published the most papers (376 papers, 10,754 citations, 28.60 citations per paper). After further analysis, we found that most institutions are more inclined to cooperate with units in their own countries.

**Table 2:** Top ten institutions’ published literature.

Rank	Institution	Country	Number of studies	Total citations	Average citation
1	Sun Yat Sen University	China	376	10,754	28.60
2	Fudan University	China	327	17,876	54.67
3	National Taiwan University	China	216	36,855	170.63
4	Zhejiang University	China	208	6,023	28.96
5	Kindai University (Kinki University)	Japan	191	32,912	172.31
6	University of California System	USA	185	30,458	164.64
7	Naval Medical University	China	182	7,948	43.67
8	Institut National de la Sante et de la Recherche Medicale (Inserm)	France	158	16,726	105.86
9	University of Hong Kong	China	149	16,402	102.51
10	Chinese Academy of Sciences	China	149	4,703	31.56

### 3.2 Journals and co-cited journals

**Figure 3:**
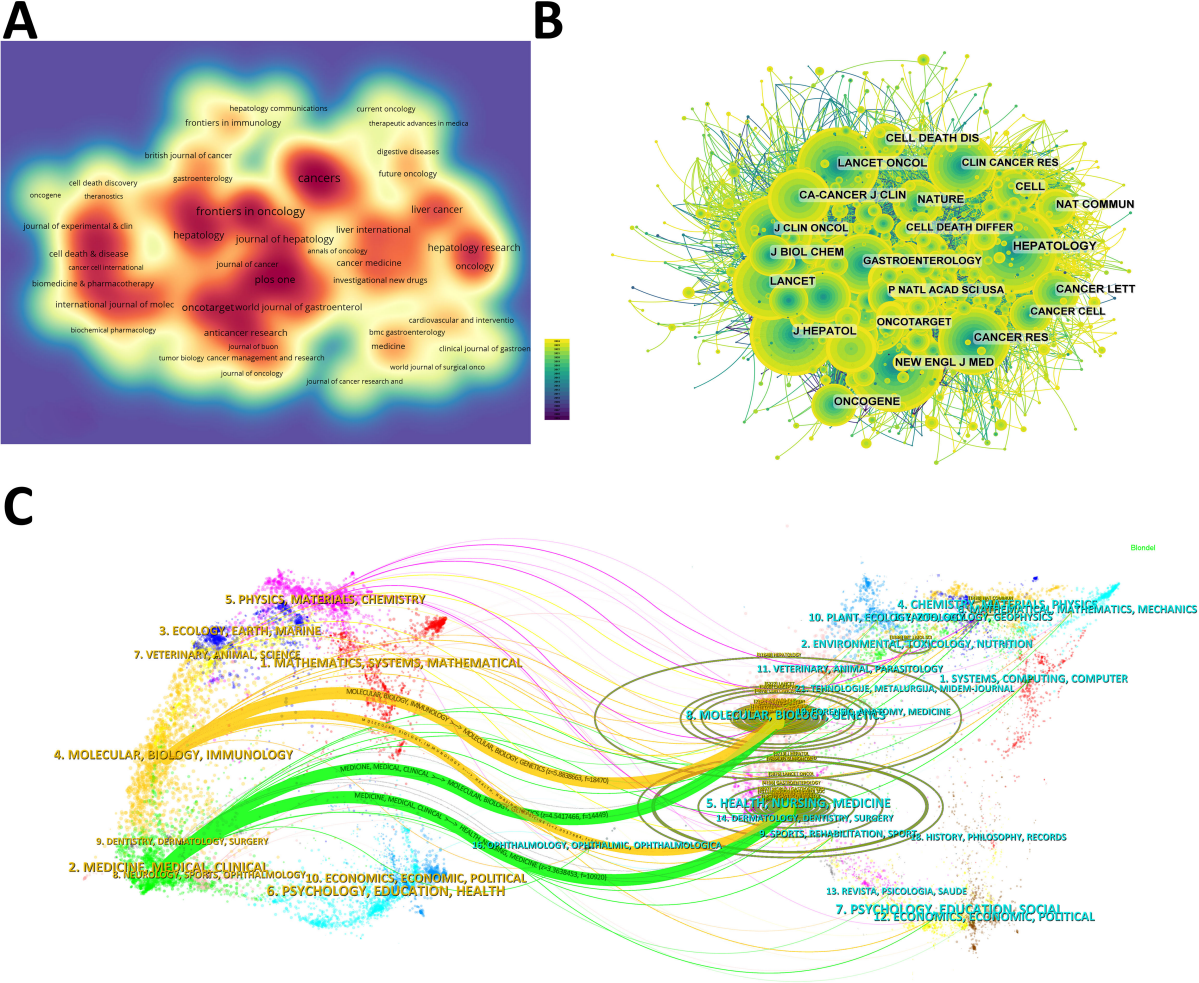
**(A)** Density map of journal publications. **(B)** Co-citation network map of journals. **(C)** Dual map of journals: the colored tracks represent citation connections, with citing journals on the left and cited journals on the right.

The density map shows the most published journals (**Figure 3A**). Then the top 10 journals with the highest output and the most citations are listed in **Tables 3**, **4**. Cancers (212 articles, 2.77%) is the journal with the most papers published in this field, followed by Frontiers in Oncology (181 articles, 2.36%), Oncotarget (139 articles, 1.81%), and Plos One (125 articles, 1.63%). Among these journals, Journal of Hepatology has the highest impact factor (IF) of 26.8. 90% of the journals are classified as Journal Citation Reports (JCR) Q1/Q2.

**Table 3:** Top ten journals’ publications.

Rank	Journal	Article counts (n=7,677, %)	Impact factor	Quartile in category
1	Cancers	212 (2.77)	4.5	Q1
2	Frontiers in Oncology	181 (2.36)	3.5	Q2
3	Oncotarget	139 (1.81)	–	–
4	Plos One	125 (1.63)	2.9	Q1
5	Scientific Reports	114 (1.49)	3.8	Q1
6	BMC Cancer	107 (1.40)	3.4	Q2
7	Liver Cancer	107 (1.40)	11.6	Q1
8	Hepatology	104 (1.36)	12.9	Q1
9	Journal of Hepatology	100 (1.30)	26.8	Q1
10	Hepatology Research	99 (1.29)	3.9	Q1

**Table 4:** Top ten co-citation of journals.

Rank	Cited journal	Co-citation	Impact factor	Quartile in category
1	N Engl J Med	5,264	96.2	Q1
2	Hepatology	4,895	12.9	Q1
3	J Hepatol	4,483	26.8	Q1
4	Lancet Oncol	3,761	41.6	Q1
5	Lancet	3,741	98.4	Q1
6	J Clin Oncol	3,619	42.1	Q1
7	Cancer Res	3,083	12.5	Q1
8	Clin Cancer Res	2,862	10.0	Q1
9	Gastroenterology	2,646	25.7	Q1
10	CA Cancer J Clin	2,556	503.1	Q1

The IF of a journal is determined by its frequency of co-citations, indicating whether the journal has a vital impact on the scientific environment. According to **Figure 3B** and **Table 4**, the journal with the most co-citations is N Engl J Med (5,264 times), followed by Hepatology (4,895 times) and J Hepatol (4,483 times). Among the top 10 journals with the most co-citations, CA Cancer J Clin was cited 2,556 times and had the highest IF among the top 10 journals (96.2). All the top 10 journals are of Q1.

The topic distribution of publications is displayed in a dual map (**Figure 3C**). Based on the displayed results, we identified two main colored citation paths: studies published in “molecular/biology/genetics” journals are mainly cited by studies published in “molecular/biology/immunology” and “medicine/medical/clinical” journals. Studies published in “health/nursing/medicine” journals are mainly cited by studies published in “molecular/biology/immunology” and “medicine/medical/clinical” journals.

### 3.3 Authors and co-cited authors

**Figure 4:**
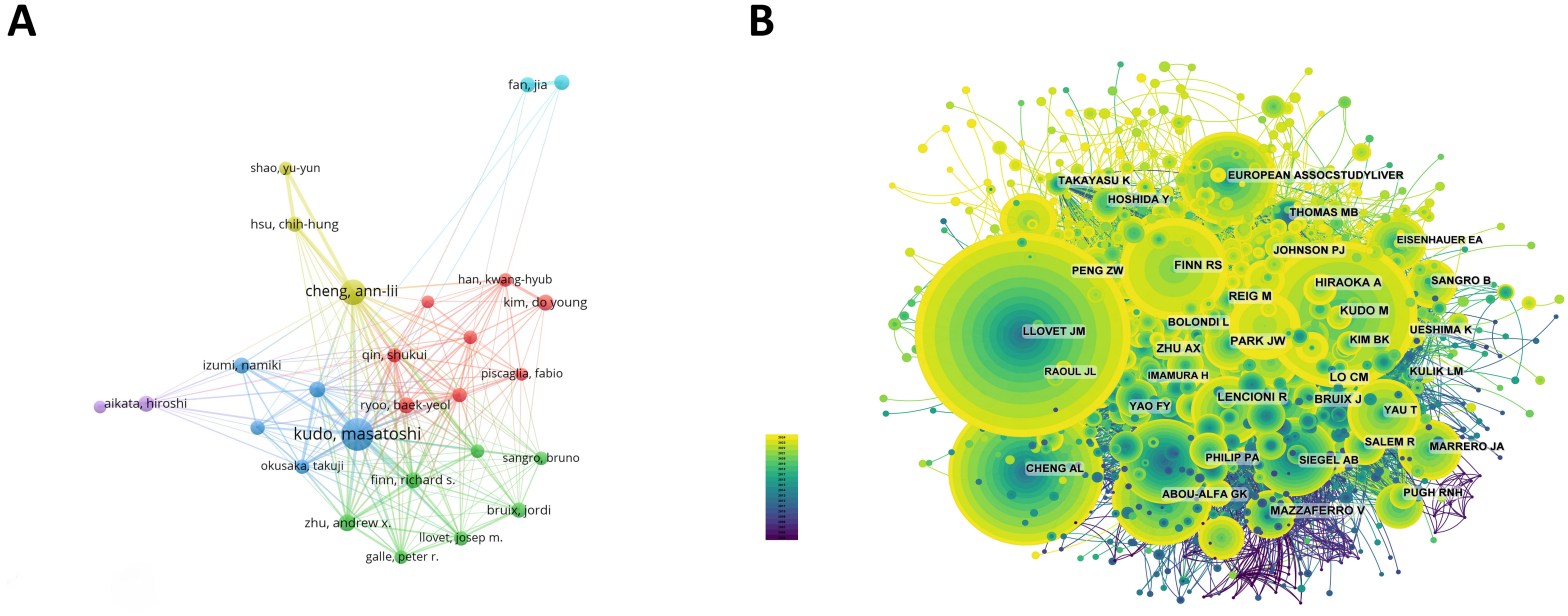
**(A)** Cooperation network of authors. **(B)** Co-citation network of authors.

Among all the authors who have published literature related to sorafenib in HCC, the top 10 who have published the most papers are listed in **Table 5**. They have published a total of 647 papers, accounting for 8.44% of all papers in this field. Kudo, Masatoshi has published the most research papers, with 141 papers, followed by Cheng, Ann-lii (101 papers) and Zhu, Andrew X. (56 papers). CiteSpace visualizes the network between authors (**Figure 4A**).

**Table 5:** Top ten authors of publications and co-citation respectively.

Rank	Author	Number of publication	Rank	Co-cited author	Citation
1	Kudo, Masatoshi	141	1	Llovet, JM	5,151
2	Cheng, Ann-lii	101	2	Bruix, J	2,869
3	Zhu, Andrew X.	56	3	Cheng, AL	2,661
4	Ryoo, Baek-yeol	52	4	Kudo, M	2,620
5	Finn, Richard S.	51	5	Finn, RS	1,485
6	Ikeda, Masafumi	50	6	Zhu, AX	1,438
7	Kim, Do young	50	7	Lencioni, R	1,288
8	Aikata, Hiroshi	49	8	Abou-alfa, GK	1,260
9	Izumi, Namiki	49	9	European Assoc Study Liver	1,098
10	Bruix, Jordi	48	10	Forner, A	1,093

The top 10 authors with the most co-citations and citations are shown respectively in **Figure 4B** and **Table 5**. 116 authors have been cited more than 50 times, indicating that their researches have a high reputation and influence. The largest nodes are associated with the authors with the most co-citations, including JM Llovet (5,151 citations), J Bruix (2,869 citations), and AL Cheng (2,661 citations).

### 3.4 Keywords co-occurrence, clusters, and evolution

**Figure 5:**
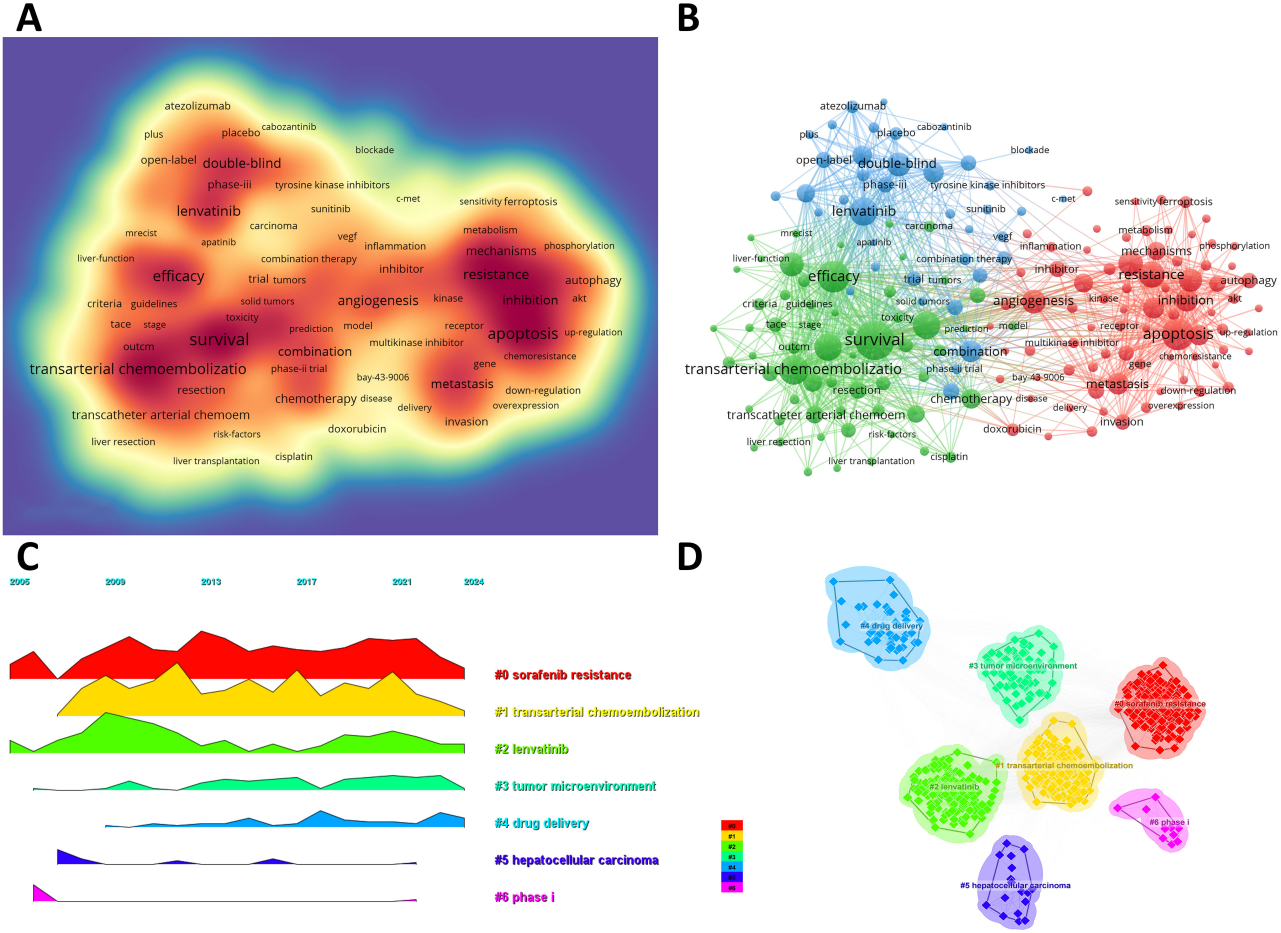
**(A)** Density map of keywords. **(B)** Network map of 178 high-frequency keywords that appeared at least 60 times; Cluster 1 (red) has 79 keywords, Cluster 2 (green) has 60 keywords, and Cluster 3 (blue) contains 39 keywords. **(C)** Peak map of keyword clustering. **(D)** Clustering map of keywords.

We can swiftly grasp the state and trajectory of this domain by examining keywords. According to the co-occurrence of keywords in VOSwiever, the most popular keyword is “survival” (1,107 times), followed by “apoptosis” (765 times), “efficacy” (745 times), “transarterial chemoembolization” (683 times) and “lenvatinib” (630 times) (**Table 6**, **Figure 5A**). We removed useless keywords and constructed a network containing 178 keywords that appeared at least 60 times, and obtained 3 different clusters (**Figure 5B**). Cluster 1 (red) has 79 keywords, Cluster 2 (green) has 60 keywords, and Cluster 3 (blue) contains 39 keywords. We used CiteSpace to draw a volcano map to visually display the changes in research hotspots over time (**Figures 5C, D**). We found that “sorafenib resistance”, “transarterial chemoembolization”, “lenvatinib”, “tumor microenvironment”, and “drug delivery” are current research hotspots.

**Table 6:** Top 20 high frequency keywords.

Rank	Keywords	Counts
1	survival	1,107
2	apoptosis	765
3	efficacy	745
4	transarterial chemoembolization	683
5	lenvatinib	630
6	prognosis	600
7	management	592
8	resistance	534
9	growth	500
10	double-blind	465
11	safety	465
12	angiogenesis	461
13	activation	451
14	inhibition	416
15	metastasis	406
16	combination	400
17	pathway	387
18	immunotherapy	368
19	proliferation	350
20	chemotherapy	342

### 3.5 Co-cited references and reference burst

**Figure 6:**
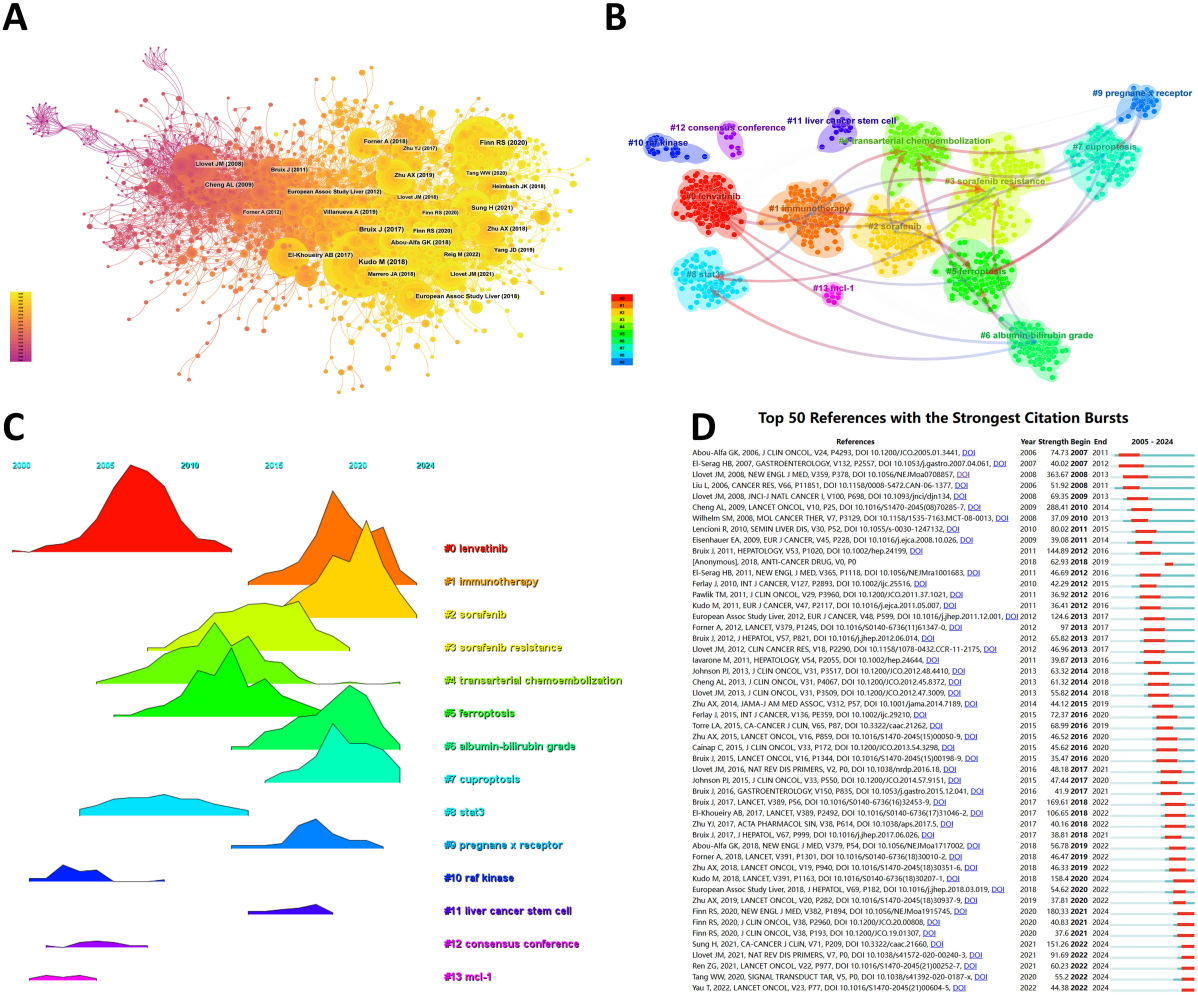
**(A)** Co-cited network of literature. **(B)** Clustering of co-cited literature. **(C)** Peak map of co-cited literature. **(D)** Bursting list of cited literature.

From 2000 to 2024, the network of co-citation references has 2010 nodes and 10,790 links (**Figure 6A**). According to the top 10 articles with the most co-citations (**Table 7**), the article by Masatoshi Kudo et al. entitled “Lenvatinib versus sorafenib in first-line treatment of patients with unresectable hepatocellular carcinoma: a randomized phase 3 non-inferiority trial” serves as the most co-citations published in Lancet (12).

**Table 7:** Top ten co-citation of literature.

Rank	Title	Journal	Author(s)	Total citations
1	Lenvatinib versus sorafenib in first-line treatment of patients with unresectable hepatocellular carcinoma: a randomized phase 3 non-inferiority trial	Lancet	Kudo M	1,530
2	Atezolizumab plus Bevacizumab in Unresectable Hepatocellular Carcinoma	N Engl J Med	Finn RS	1,080
3	Regorafenib for patients with hepatocellular carcinoma who progressed on sorafenib treatment (RESORCE): a randomized, double-blind, placebo-controlled, phase 3 trial	Lancet	Bruix J	934
4	Sorafenib in advanced hepatocellular carcinoma	N Engl J Med	Llovet JM	632
5	Global cancer statistics 2020: GLOBOCAN estimates of incidence and mortality worldwide for 36 cancers in 185 countries	CA Cancer J Clin	Sung H	553
6	Efficacy and safety of sorafenib in patients in the Asia-Pacific region with advanced hepatocellular carcinoma: a phase III randomized, double-blind, placebo-controlled trial	Lancet Oncol	Cheng AL	537
7	Cabozantinib in Patients with Advanced and Progressing Hepatocellular Carcinoma	N Engl J Med	Abou-Alfa GK	533
8	Nivolumab in patients with advanced hepatocellular carcinoma (CheckMate 040): an open-label, non-comparative, phase 1/2 dose escalation and expansion trial	Lancet	El-Khoueiry AB	530
9	EASL Clinical Practice Guidelines: Management of hepatocellular carcinoma	J Hepatol	European Assoc Study Liver	478
10	Ramucirumab after sorafenib in patients with advanced hepatocellular carcinoma and increased α-fetoprotein concentrations (REACH-2): a randomized, double-blind, placebo-controlled, phase 3 trial	Lancet Oncol	Zhu AX	452

Then, we performed co-citation reference clustering and time clustering analysis (**Figures 6B, C**). We found that “lenvatinib” (cluster 0), “raf kinase” (cluster 10), “consensus” (cluster 12), and “mcl-1” (cluster 13) are early research hotspots, and “stat3” (cluster 8) is a mid-term research hotspot; “immunotherapy” (cluster 1), “sorafenib” (cluster 2), “sorafenib resistance” (cluster 3), “transarterial chemoembolization” (cluster 4), “ferroptosis” (cluster 5), “albumin bilirubin grade” (cluster 6), “cuproptosis” (cluster 7), “pregnancy x receptor” (cluster 9), and “liver cancer stem cell” (cluster 11) are hot topics and current trends in this field.

Through CiteSpace, we obtained the 50 most reliable citation bursts of sorafenib in HCC research. The reference with the highest citation rate (18.64) is "Sorafenib in Advanced Hepatocellular Carcinoma" by Josep M. Llovet et al. and published in N Engl J Med (13). All these 50 references were published between 2005 and 2024, indicating that these papers have been frequently cited in the past 20 years. Importantly, 9 of these papers are currently at their peak of citations (**Figure 6D**), which means that sorafenib will continue to receive attention in the field of HCC research.

## 4 Discussion

To our knowledge, this is the first bibliometric analysis to investigate the application of sorafenib in HCC, which focused on analyzing scholarly publications, pinpointing dominant countries and institutions, gauging global cooperations, exploring research hotspots, and spotting surges in keyword and citation bursts. A total of 7,667 articles related to sorafenib in HCC were included from 2005 to 2024. Serving as the first approved TKI drug of HCC targeted therapy in 2007, sorafenib is still a benchmark for HCC precision treatment (13).

From the year of 2009, the number of articles in this field increased rapidly, with the highest number in 2022. This trend indicates that sorafenib received extreme attention for HCC during this period. The rapid development may be mainly the results of (I) the increasing screened number of HCC patients especially in developing countries; (II) the rapid attention of personalized therapy of advanced HCC worldwide; and (III) the high-quality international collaborations on the clinical trials in this field (2, 14). As the biggest country with Hepatitis B virus infection, China included sorafenib for HCC in its medical insurance in 2017 (15). This is consistent with our study, which visually shows that the growth rate has exploded since 2017. It provided favorable conditions for this country to carry out large-scale clinical researches. Although most countries worldwide have initiated their researches on this field, we found in our study that there is a lack of mutual cooperations with different countries and institutions, even in the top ones. Therefore, we call for strengthening cooperation between domestic and foreign institutions to break down academic barriers, so as to contribute to the long-term development of this field.

In our study, we focused on the hotspots and frontiers in the sorafenib-HCC field. Based on the analysis of keywords and co-cited references as well as their bursts, we found that the research focus of sorafenib-HCC is closely related to (I) the apoptosis of HCC, (II) the clinical trials of advanced HCC, and (III) the combination treatment of HCC.

As noted earlier, sorafenib can induce HCC cell apoptosis by promoting cellular autophagy (5). Further studies revealed that alterations in autophagy are associated with more aggressive behavior of HCC cells and their reduced sensitivity to sorafenib (16). Autophagy is a process of cellular self-digestion that plays a crucial physiological role in the liver, and autophagy dysregulation has even been associated with HCC (17, 18). However, autophagy can drive drug resistance or sensitivity, act as a double-edged sword (6, 19). Studies indicated that the combining use of sorafenib and autophagy modulators can regress chemotherapy resistance, which is characterized by lower VEGFa and HIF-1α at the level of gene transcription, and inhibition of Gal-1 and NF-κB protein expression (6). These interesting phenomena have aroused interest in the deeper exploration of autophagy in sorafenib-HCC field. For example, a recent study by He et al. explored the trends and hotspots of autophagy in HCC, which highlighted that autophagy-related pathways, proteins, genes and targets to overcome sorafenib resistance have emerged as prominent focal points in recent research on autophagy in HCC (20). Therefore, future studies will also be investigating ways to modulate autophagy (autophagy promoters or inhibitors) to improve the effectiveness of anticancer treatments or address resistance to current HCC therapies.

In our bibliometric analysis, the REFLECT study by Masatoshi Kudo et al. (NCT01761266) was co-cited most (12), followed by Richard Finn et al.’s IMbrave150 study (NCT03434379) (21). The REFLECT study indicated that lenvatinib is not inferior to sorafenib in advanced HCC patients’ overall survival (13.6 months vs. 12.3 months) with a similar safety and tolerability. The comparison between lenvatinib and sorafenib is indeed a hotspot of our study. As the first-line treatment, sorafenib resistance usually develops within 6 months among advanced HCC patients (22). As a result, apart from sorafenib and lenvatinib, multiple TKI drugs for HCC and other malignancies have been applied during the past decade, and the patients’ survival has been further upgraded. Moreover, In IMbrave150 study, Richard Finn et al. revealed that atezolizumab plus bevacizumab reached better response rate, progression-free survival (6.8 months vs. 4.3 months), overall survival (one-year rate: 67.2% vs. 54.6%), and quality of life in first-line advanced HCC patients, compared to sorafenib alone. These findings not only stimulate new drug developing, but also call for comprehensive treatment for HCC (23). For example, while sorafenib remains the first-line therapy in many regions, the introduction of newer agents like lenvatinib has shifted treatment paradigms, fostering competition and innovation. One ongoing trial explored sorafenib's role in adjuvant settings to prevent HCC recurrence after surgical resection or ablation (24). This report shows the changes in the researches and clinical applications of sorafenib in HCC.

In our study, some other top hotspots lie in the field related to immunotherapy and transarterial chemoembolization (TACE), etc. The combinations of sorafenib with other drugs or local therapies have been constantly investigated to circumvent the limitations of sorafenib in HCC patients (25). As the representative immunotherapeutic drug, immune checkpoint inhibitor (ICI) shows powerful efficacy in late-stage HCC (21). When combined with ICI immunotherapy, TKI drugs can downregulate certain molecules’ levels and inhibits the corresponding pathways, thereby overcoming HCC resistance to ICI therapy (26–28). TACE is the standard treatment of mid-stage HCC, and it shows better efficacy when combined with sorafenib compared with sorafenib alone for patients with Barcelona Clinic Liver Cancer B/C stages (23, 29). However, due to the heterogeneity of tumors, physical status and different sensitivity to drugs, the prognosis of individuals varies. Therefore, TACE combined with targeted therapy has a poor effect on some patients, and long-term use of this treatment regimen for such patients may increase the financial burden and even shorten their survival. Still, there is a lack of molecular predictors that can predict the efficacy of sorafenib for HCC (30). Therefore, this is another research orientation in the future. Furthermore, combining sorafenib with other pharmacological agents has shown promise in preclinical and clinical studies. Ursodeoxycholic acid, for example, synergistically enhances sorafenib's antitumor activity by modulating the STAT3 and ERK signaling pathways (31). This combination inhibits tumor proliferation and promotes apoptosis, offering a potential strategy for advanced HCC. Similarly, berbamine and ouabain, inhibitors of Na^+^/K^+^-ATPase, amplify the efficacy of sorafenib by disrupting ion homeostasis and inducing cell death, highlighting an innovative approach for therapy (32). As there are still limited drugs or drug combinations showing better effectiveness compared to sorafenib in advanced HCC, we believe that the combinations of sorafenib with more novel drugs (e.g. immunologic and synergistic drugs) or local therapies (e.g. infusion chemotherapy and radiofrequency ablation) will continue to be the research hotspots in the future.

By analyzing the keywords of this study, we found “sorafenib resistance” and “drug delivery” are another two research hotspots (**Figure 5**). Despite autophagy, immune-mediated factors, the tumor microenvironment (TME), cancer stemness and other epigenetic regulators (e.g. microRNAs) also play a pivotal role in sorafenib resistance (33, 34). To overcome sorafenib resistance, multiple methods are performed according to these mechanisms. For example, targeting enzymes in the tumor protein pathways (e.g. phosphoglycerate dehydrogenase, PHGDH) is an effective method to avoid sorafenib resistance (35). Furthermore, drug delivery systems, especially nanodrug delivery have revolutionized cancer therapy (36). By addressing the complexity of the TME and circumventing drug resistance pathways, nanodrugs offer significant potential to enhance sorafenib efficacy (37). For example, Ubiquitin-specific protease 22 (USP22) is recognized for its critical role in promoting HCC stemness and contributing to multidrug resistance, thus a galactose-decorated lipopolyplex (Gal-SLP) has been designed as a targeted, cascade-responsive nanoplatform for HCC, enabling the co-delivery of sorafenib and USP22 shRNA to achieve synergistic therapeutic effects (38). Therefore, drug delivery systems like Gal-SLPs demonstrate strong antitumor efficacy and high biosafety, making them a promising candidate for the clinical treatment of HCC.

In our study, we found that the research interest shows regional differences globally. Asia is the leading region in the number of related publications, of which China and Japan are the core countries. Sorafenib has been widely used around the world and its price is relatively high, but it has been included in the medical insurance of many countries. However, in regions with limited healthcare resources, challenges such as its high cost and significant side effects necessitate targeted approaches to ensure equitable access. Donafenib, also known as a similar TKI drug as sorafenib, is relatively cheaper and more easily included in China's medical insurance system (39). Therefore, the research depth of sorafenib in China may be no major breakthrough in the future, and this is also a reason why there is a probably stagnation of the publication of sorafenib in HCC over the past two years (**Figures 2A, B**). A deeper investigation into sorafenib’s economic and therapeutic impact, especially in regions with limited healthcare resources, could provide actionable insights for improving HCC treatment standards universally.

Despite the promising findings and research trends, the study acknowledges several limitations. One significant limitation is only one single WoSCC database was used, which may exclude relevant publications indexed in other databases or those not included in WoSCC, thereby result in some missing but important publications. Second, non-English publications were excluded, which may have led to the omission of valuable researches from non-English-speaking regions such as Asian countries. Third, our output results might exist biases due to certain changes in the names of authors and institutions. Nevertheless, this bibliometric analysis can help scholars further understand the hotspots in sorafenib-HCC field and make a significant contribution to the literature.

## 5 Conclusions

To the best of our knowledge, this is the first bibliometric analysis in sorafenib-HCC field. We identified current hotspots and trends in this field, which might provide valuable guidance for future researches. Further explorations are supposed to conduct the continued study of HCC apoptosis, large-scaled clinical trials with international cooperations, and comprehensive treatments including multiple systemic or locoregional approaches in patients with HCC.

## Data Availability

The original contributions presented in the study are included in the article/supplementary material. Further inquiries can be directed to the corresponding authors.
